# Essentials of Diagnosis, Arranged in the Form of Questions and Answers

**Published:** 1900-11

**Authors:** 


ESSENTIALS OF DIAGNOSIS, Arranged in the Form of Questions and Answers. Prepared especially for students of medicine, by Solomon Solis Cohen, M. D., Professor of Clinical Medicine and Therapeutics in the Philadelphia Polyclinic, Etc., and Augustus A. Eshner, M. D., Professor of Clinical Medicine in the Philadelphia Polyclinic, Etc. Illustrated. Second edition, revised and enlarged. Pages, 417; price, $1.00. Philadelphia: W. B. Saunders, 925 Walnut Street. 1900.

This embraces the subject of diagnosis in small compass. One in a hurry to get through a great deal of work in a very short time, this book will greatly aid. It is not intended to supplant the more extensive text-book, but the student who finds it necessary to "cram" for examination will certainly find a friend in Cohen and Eshner's Essentials of Medical Diagnosis.

